# Cross-Antigenicity between EV71 Sub-Genotypes: Implications for Vaccine Efficacy

**DOI:** 10.3390/v13050720

**Published:** 2021-04-21

**Authors:** Pei Liu, Yadi Yuan, Bopei Cui, Yaqian Huo, Lianlian Bian, Lei Chen, Siyuan Liu, Chenfei Wang, Yingzhi Xu, Alison Tedcastle, Fan Gao, Qunying Mao, Javier Martin, Zhenglun Liang

**Affiliations:** 1 National Institutes for Food and Drug Control, Beijing 102600, China; liupei2021@163.com (P.L.); yuan95207@163.com (Y.Y.); cbp31@139.com (B.C.); huoyaqian1020@126.com (Y.H.); bianlian2015@126.com (L.B.); zjyxiaoliu@163.com (S.L.); c.f.wang@hotmail.com (C.W.); lzhenglun@126.com (Z.L.); 2 Minhai Biotechnology CO., LTD, Beijing 102600, China; wxhwxxs@163.com (L.C.); xuyingzhi@biominhai.com (Y.X.); 3 Division of Virology, National Institute for Biological Standards and Control, Potters Bar, Hertfordshire EN6 3QG, UK; alison.tedcastle@nibsc.org (A.T.); Javier.Martin@nibsc.org (J.M.)

**Keywords:** enterovirus 71 (EV71), vaccine, cross-neutralization, genotype

## Abstract

Enterovirus A-71 (EV71) is a global, highly contagkkious pathogen responsible for severe cases of hand-food-mouth-disease (HFMD). The use of vaccines eliciting cross neutralizing antibodies (NTAbs) against the different circulating EV71 sub-genotypes is important for preventing HFMD outbreaks. Here, we tested the cross-neutralizing activities induced by EV71 genotype/sub-genotype A, B0-B4, C1, C2, C4, and C5 viruses using rats. Differences were noted in the cross-neutralization of the 10 sub-genotypes tested but there were generally good levels of cross-neutralization except against genotype A virus, against which neutralization antibody titres (NTAb) where the lowest with NTAbs being the highest against sub-genotype B4. Moreover, NTAb responses induced by C4, B4, C1, and C2 viruses were homogenous, with values of maximum/minimum NTAb ratios (MAX/MIN) against all B and C viruses ranging between 4.0 and 6.0, whereas MAX/MIN values against B3 and A viruses were highly variable, 48.0 and 256.0, respectively. We then dissected the cross-neutralizing ability of sera from infants and children and rats immunized with C4 EV71 vaccines. Cross-neutralizing titers against the 10 sub-genotypes were good in both vaccinated infants and children and rats with the MAX/MIN ranging from 1.8–3.4 and 5.1–7.1, respectively, which were similar to those found in naturally infected patients (2.8). Therefore, we conclude that C4 EV71 vaccines can provide global protection to infants and children against HFMD caused by different sub-genotypes.

## 1. Introduction

Enterovirus 71 (EV71) is a 30 nm spherical, non-enveloped virus, belonging to the *Enterovirus* genus of the Picornaviridae family. The viral genome is made up of an approximately 7.5 kb positive single-stranded RNA [1]. Although EV71 includes only one serotype, eight genotypes (A-H) have been identified so far, with genotypes B and C, including sub-genotypes B1-B5 and C1-C5 being the most commonly circulating and associated with disease [2]. In recent years, new genotypes D-H have been reported in India, Pakistan, and Africa but information on the extent of virus transmission and association with disease is lacking [3,4]. Human infections of EV71 result in hand-foot-mouth-disease (HFMD), which is characterized by fever, sore throat, skin rashes, and mouth sores. Although most of the patients recover on their own within 1–2 weeks, complications like viral encephalitis, brain stem encephalitis, pulmonary edema, and acute flaccid paralysis or even death have been reported in minor infants and children [5,6]. Since the isolation and identification of the first strain of EV71 in 1969 from the Americas [7], numerous outbreaks of the virus have been reported worldwide [8,9,10,11,12,13,14]. The virus has become highly prevalent in the Western Pacific region after the outbreak in Taiwan in 1997, persistently causing seasonal outbreaks with enhanced severity and mortality [15]. In 2008, HFMD was listed as a category-C infectious disease in China. Between 2009 and 2016, 1.16–1.92 million cases of HFMD were documented with a mortality rate of 0.03–0.05% [16]. After poliovirus, EV71 has become the most serious threat ever posed by enteroviruses as a neurotrophic agent [17].

Vaccination is the most economical and effective method to prevent and control the spread of infectious diseases. Three kinds of EV71 inactivated vaccines were approved in December 2015 in mainland China, making China the only country using vaccines to prevent HFMD [18]. All the three vaccines are based on the C4 sub-genotype of EV71. The vaccines have been proved to be more than 90% effective in clinical trials involving the participation of 30,000 infants and children [19,20,21]. Notably, vaccination almost completely prevented the manifestation of severe symptoms. Subsequently, EV71 vaccines have been administered to 40 million individuals considered to be at a higher risk of contracting HFMD. The vaccination has been carried out across most of the regions of China, leading to a significant drop in HFMD-associated mortality. The death toll decreased 92.7% and 95.8% in 2018 and 2019, respectively, compared with the numbers preceding vaccine approval [22].

Incidences of mutations and recombination are known to occur during outbreaks of EV71. By virtue of co-circulation and co-infection with other serotypes and genotypes, viral RNA can get assorted between different strains, contributing to the continuous change in the genetic makeup of prevalent strains. In line with this, sub-genotypes of EV71 continue to evolve [23]. The earliest genotype, for instance A (BrCr strain), was isolated in 1969 in California, while sub-genotype B0 was identified in the 1960s. This sub-genotype became the prevalent strain along with genotype A [24]. B1 and B2 sub-genotypes caused epidemics in Europe and America in the 1970s and 1980s, respectively, and abundant B1 and B2 strains were also isolated from HFMD patients in Australia and Japan [25,26,27,28,29]. The C1 sub-genotype caused pandemics in Europe and the United States in the late 1980s, and the C2 sub-genotype and C1 sub-genotype have alternated in Europe and America since 1995 [23]. After 1997, HFMD was mainly prevalent in the Asia Pacific region and various sub-genotypes such as B3~B5 and C3~C5 appeared in less than 20 years [30,31,32,33,34,35]. In mainland China, the sub-genotype C4 continues to remain the predominance since the first EV71 strain isolated in 1997 [2].

In order to further understand the cross-antigenic properties of different circulating EV71 genotypes/sub-genotypes we have analyzed the cross-neutralization activities using rats inoculated with different individual viruses. In addition, we have analyzed sera collected from rats and humans immunized with C4 EV71 vaccines in clinical trials for cross-neutralization activity against various sub-genotypes of EV71 and compared them with the values obtained against the C4 sub-genotype viruses used in the formulation of the vaccines. The results are discussed in terms of the role of EV71 vaccines for the global prevention and control of EV71 induced severe-HFMD.

## 2. Materials and Methods

### 2.1. Serum Samples

#### 2.1.1. Collection of Serum Samples from Phase-Ⅳ Clinical Trial Participants Vaccinated with EV71 Vaccines

Serum samples were collected from 140 individuals who took part in phase Ⅳ clinical studies of inactivated EV71 vaccines produced by Sinovac Biotech Company (ClinicalTrials.gov 17 April 2020. identifier: NCT03909074), Wuhan Institute of Biological Products Co. Ltd. (CTR20191004), and Institute of Medical Biology Chinese Academy of Medical Science (ClinicalTrials.gov 17 April 2020. identifier: NCT03909074) (40 sera per manufacturer). These three vaccines were blinded as Manufacturer A, B, C. The sera samples collected were evaluated for cross-reactive NTAb titers against 10 sub-genotypes of EV71. Serum samples from 120 participants who received two doses of inactivated EV71 vaccine on days 0 and 30 were tested in this study. The donors were divided into two subgroups according to their baseline NTAb level against EV71 before vaccination. The participants with baseline NTAb titer less than 1:8 were categorized as the initially seronegative group who were considered to be uninfected cases (20 serum samples per manufacturer), and the remaining participants with NTAb titer more than 1:8 were categorized as the initially seropositive group who were considered to be sub-clinically infected cases because they were all infants and children without any history of HFMD (20 serum samples per manufacturer). This study also included 20 sub-clinically infected cases which were from infants and children without the history of HFMD and were not vaccinated. These infected cases were categorized as natural infection group. Written informed consent was received from donors’ guardians.

#### 2.1.2. Collection of Serum Samples from Rats Inoculated with Different Sub-Genotypes of EV71

SPF level Wistar rats were intraperitoneally (i.p.) inoculated with each of the 10 subgenotype of EV71(10^7.69^–10^9.53^CCID_50_ of viruses per rat, two rats per virus) listed in Table 1 separately twice or three times at intervals of 14 days and then bled seven days after immunization for the collection of serum samples for evaluation of their neutralization activities.

#### 2.1.3. Serum Samples from EV71 Vaccine-Immunized Rats

SPF level Wistar rats were i.p. immunized with the three types of C4 EV71 vaccines (human vaccinated dosage per rat, six rats per vaccine) separately twice at days 0 and 14 and then bled at day 21 post-immunization for the collection of serum samples for evaluation of their neutralization activities. All animal experiments were approved by the National Institute for Food and Drug Control (NIFDC) prior to the commencement of the studies.

### 2.2. Cells and Viruses

EV71 virus A (BrCr) was purchased from ATCC. EV71 virus B0–B4, C1, C2, and C5 were kindly available from stocks at the National Institute for Biological Standards and Control (NIBSC, London, UK) while virus 523-07T/C4 was propagated from samples of the virus preserved at NIFDC. All viruses were propagated in rhabdomyosarcoma (RD) cells using MEM solution (GIBCO, Gaithersburg, MD, USA containing 2% (*v*/*v*) fetal bovine serum (GIBCO), 2 mM l-glutamine (GIBCO), and 100 IU/mL penicillin and streptomycin (GIBCO). The 50% tissue culture infectious doses (TCID_50_) were determined by using RD cells, and the values were calculated using the Reed–Muench method. Details of the viral strains used in the study are showed in Table 1.

### 2.3. Sequence Analyses

P1 and VP1 regions of the 10 sub-genotypes of EV71 were analyzed. Total RNA was extracted from 50 μL of viral cultures using kits and following the manufacturer’s instructions. One-step RT-PCR amplifications were conducted, and the segments were sequenced by Delivectory Biosciences Inc, Beijing, China. Nucleotide and amino acid sequences were analyzed by MEGA7.0 (MEGA Software Development Team). A maximum-likelihood tree was constructed including 245 sequences from genebank and 10 sequences in this study by Figure Tree 1.4.4 (open access) and Adobe illustrator CC 2019 (Adobe, San Jose, CA, USA).

### 2.4. CPE Assays for the Detection of NTAb against Different EV71 Sub-Genotypes

A conventional CPE assay was employed to measure NTAb titers against different EV71 sub-genotypes. Blood samples were inactivated at 56 °C for 30 min, and serially diluted two-fold from 1:8. A total of 50 μL serially diluted sera and 50 μL virus preparation containing 100 TCID_50_ of EV71 were mixed in 96-well microplates and incubated with RD cells. CPE were observed using inverted microscope after incubation for seven days. NTAb titers of EV71 were confirmed when RD showed 50% inhibition of CPE. Samples were run simultaneously with wells of cell control, positive serum control, and virus back titration. NTAb titers equivalent to or more than 1:8 were assigned as sero-positive, while those less than 1:8 were considered sero-negative.

### 2.5. Statistical Analysis

Excel 2020, SPSS Statistics 21 and Graphpad Prism v8.0 were utilized to perform statistical processing and analyses of the data. Geometric mean titer (GMT) and their 95% confidence intervals (95% CI) were calculated, converted to the logarithmic scale and analyzed by using one-way ANOVA, considering *p* < 0.05 as a statistically significant difference.

## 3. Results

### 3.1. Cross-Neutralization Activities of Sera from Rats Inoculated with EV71 Virus Strains from 10 Different Genotypes/Sub-Genotypes

Rats were inoculated with 10 sub-genotypes of EV71 individually (Table 1). Sera from inoculated animals were tested for their cross-neutralizing activity against each of the 10 EV71 sub-genotype viruses. Results of the cross-neutralization assays revealed that genotype A EV71 virus could be neutralized by sera raised against EV71 A genotype and EV71 B1, B2, B4, C4, and C5 sub-genotypes only (Table 2). In contrast, type B and C genotypes could be neutralized by all anti-sera. However, there were differences in the efficiency of cross-neutralization. GMT against EV71 A was 14.3, significantly lower than GMTs elicited against other EV71 sub-genotypes (all *p* < 0.05). The GMT values for B1 (65.5) and B0 (78.4) were better than those against A. The GMT values of sera against B2, B3, C4, and C5 were between 115.4–178.0. The highest GMT value was recorded against B4 (313.5). These results indicated that the strain of B4 was the most readily neutralized by sera from rats inoculated by various sub-genotypes of EV71, followed by B2, B3, C4, and C5. Interestingly, the A genotype virus was the least well neutralized, which may be related to the fact that it is the most genetically distant from other EV71 sub-genotypes. Taking into consideration the facts that (1) the cross-neutralization efficiency of NTAbs elicited by rats inoculated with A was significantly lower than those induced by other sub-genotypes; and (2) there have been no epidemics caused by A genotype anywhere in the world for a long time, the subsequent analysis of cross-neutralization efficiencies of sera were carried out with and without A.

The cross-neutralizing capacity of sera against different sub-genotypes of EV71 was analyzed. The difference between the cross-neutralization abilities of each serum sample was represented by comparing the ratio between maximal NTAb and minimal NTAb values (MAX/MIN) against the 10 EV71 sub-genotypes. These values reflected the range of cross-neutralization activities elicited by each sub-genotype in rats. The results showed that, except for A, the MAX/MIN ratios of all other sera against 9 EV71 sub-genotypes was in the range of 4.0–256.0. Within these B4, C1, C4, and C2 elicited a fairly homogenous response with the MAX/MIN ratios being 4.0, 4.0, 5.3 and 6.0, respectively; MAX/MIN values for sera induced by B0, C5, B1, and B2 were 10.7, 10.7, 12.0, 16.0, and 48.0, respectively; the neutralizing titer of A anti-serum against the strains of nine sub-genotypes was the maximal with a MAX/MIN value of 256.0. The MAX/MIN ratios of all sera against the 10 sub-genotypes fell in the range of 6.0–384.0 when A sub-genotype was included. Compared to the data with A, the MAX/MIN ratio of B2 anti-serum increased from 16 to 384 after excluding data of A (Figure S1), whereas MAX/MIN of other B and C antisera increased 1.3–6 times.

### 3.2. Cross-Neutralizing Activities of Sera from Rats Inoculated with C4 EV71 Inactivated Vaccines

Rats were vaccinated with three different C4 EV71 vaccines. Sera from immunized animals were used for cross-neutralization assays using the strains of 10 genotypes/sub-genotypes EV71.Results showed that NTAb GMTs induced by vaccines A, B, and C against the 10 EV71 genotype/sub-genotypes fell in the ranges 37.4–627.1, 172.9–5325.2, and 805.7–3649.1, respectively (Table 3).

NTAb GMT against A was 173.4, which was significantly lower than against B0, B1, B2, and C5 EV71 sub-genotypes (*p* < 0.05), indicating that A genotype was the most poorly neutralized, consistent with the cross-neutralization results of sera from rats inoculated with viruses. The GMT of sera against C5 was maximal (GMT = 2165.0), indicating that C5 was the best neutralized by vaccine-immunized sera. GMT against C4 (GMT = 448.6) was only higher than that of A, which was contradictory to the result of serum against 523-07T/C4, indicating that cross-neutralizing antibody elicited by different strains of C4 might be discrepant. The aforementioned results indicated that C4 EV71 vaccines could induce a broad spectrum of cross neutralizing antibodies against A, B, and C genotypes in experimental animals and manifested better cross-neutralizing capacity against B and C genotypes compared to the challenge strain of C4.

The homogeneity of the immune response induced by the three vaccines against all strains was analyzed. Results showed that the values of NTAb MAX/MIN ratios including values against genotype A for vaccines A, B, and C were 16.8, 30.8, and 5.1, respectively; the values of NTAb MAX/MIN ratios without values against genotype A for vaccines A, B, and C were were lower, 7.1, 6.3, and 5.1, respectively, in agreement with the results obtained in the viral challenge studies.

Overall, sera of animals immunized with the three vaccines manifested good cross-neutralizing activities against B and C genotypes (MAX/MIN = 5.1–7.1) although some significant differences were found between NTAbs against different EV71 sub-genotypes.

### 3.3. Cross-Neutralizing Activity of Sera from Infants and Children Immunized with C4 EV71 Inactivated Vaccines

We explored the cross-neutralizing ability of sera from clinical trials against different EV71 sub-genotypes. Sera from vaccines being sero-negative or sero-positive before immunization with vaccines A, B and C (20 samples per manufacturer) and sera from naturally infected infants were included in this study. Three types of sera were used to compare their cross-neutralizing activities against the 10 EV71 genotypes/sub-genotypes. As shown in Table 4, the results showed that the GMTs of sera from natural infected individuals against A, B, and C genotypes were in the range of 40.8–724.6; GMTs of sera from initially sero-positive individuals against A, B, and C genotypes were 113.1–1688.7, 183.8–3589.3, 205.1.3–4874.3, respectively; GMTs of sera from initially sero-negative individuals against A, B, and C genotypes were 15.3–400.7, 32.0–463.0, and 42.8–549.1, respectively. NTAb GMTs against EV71 A were significantly lower than those against other EV71 sub-genotypes (*p* < 0.05 in all groups), regardless of the source of the sample. The results indicated that both human and animal sera had the weakest neutralization capacity against the genotype A EV71 strain.

The results were further analyzed without data against genotype A. The GMTs of sera from naturally infected population against B and C genotypes were between 256.2–724.6 and the MAX/MIN was 2.8; GMTs of sera from initially sero-positive population against B and C genotypes were between 701.7–1688.7 (vaccine A), 1862.8–3589.3 (vaccine B), 2681.3–4874.3 (vaccine C) and their corresponding MAX/MIN ratios were 2.4, 1.9, and 1.8, respectively; GMTs of sera from initially sero-negative population against B and C genotypes were 118.5–400.7 (vaccine A), 199.8–463.0 (vaccine B), 304.6–549.1 (vaccine C) and their corresponding MAX/MIN ratios were 3.4, 1.8, and 1.8, respectively. The MAX/MIN ratios of sera from vaccinated individuals were clearly lower than those of sera from animals inoculated with viruses. The antibodies of initially sero-negative population were considered to be induced by vaccination, which were more meaningful than those of initially sero-positive population. The MAX/MIN ratios of initially sero-negative population were 1.8–3.4, suggesting that sera from C4 vaccinated individuals showed broad cross-neutralizing ability against B and C genotypes, which were close to values observed with sera from naturally infected individuals.

**Figure 1:**
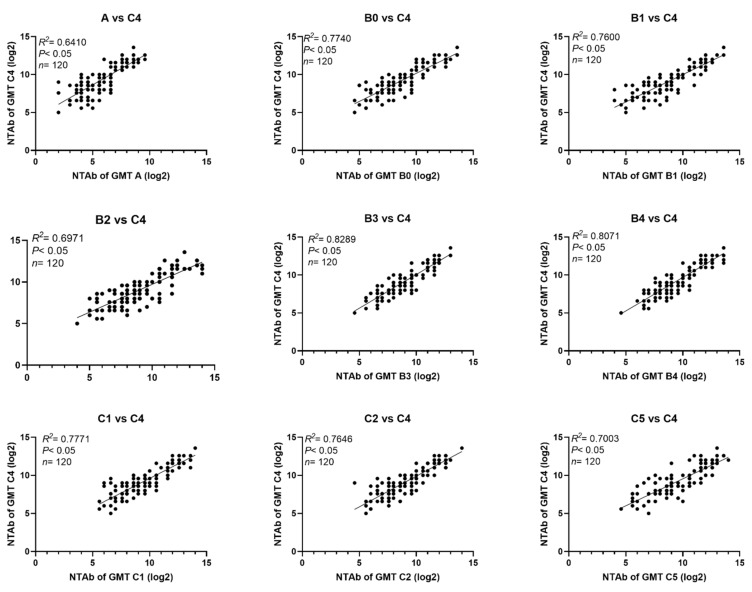
Correlation between NTAbs against different EV71 sub-genotypes and those against sub-genotype C4 in sera from infants and children immunized with vaccines.

**Figure 2:**
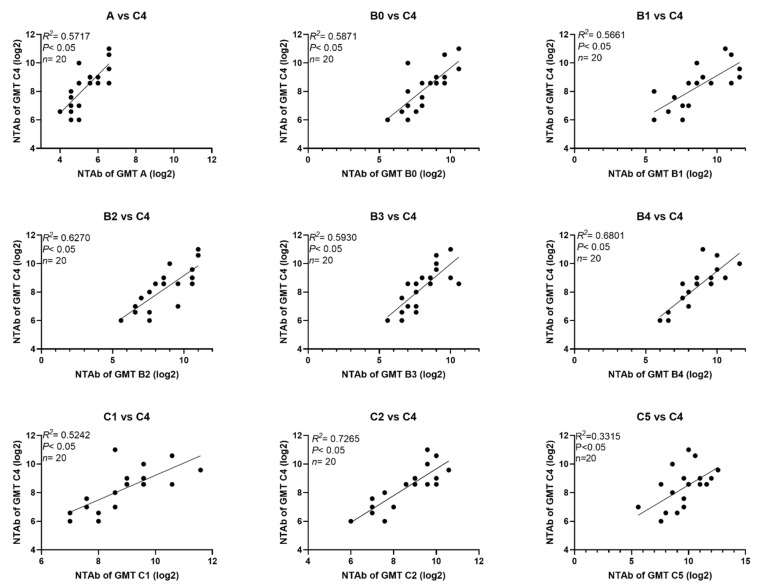
Correlation between NTAbs against different EV71 sub-genotypes and those against sub-genotype C4 in naturally infected infants and children.

NTAbs against the 523–07T/C4 strain were used for the evaluation of the potency of the EV71 vaccines in clinical trials [36]. For this reason, we analyzed the correlation of NTAbs against the different genotypes/subgenotypes relative to those against virus 523-07T/C4 in sera from vaccinated and naturally infected infants and children (Figure 1 and Figure 2). The results showed good correlation between NTAb values against A, B0-B4, C1, C2, C5 viruses, and those against 523-07T/C4 virus in sera from vaccinated infants and children with R^2^ values 0.6410, 0.7747, 0.7600, 0.6971, 0.8289, 0.8071, 0.7771, 0.7646, and 0.7003, respectively. Inferior correlation was found between NTAb values against A, B0-B4, C1, C2, and C5 viruses relative to those against 523-07T/C4 virus in sera from naturally infected infants and children, particularly between NTAb titers against C5 and C4 sub-genotypes with R^2^ values 0.5717, 0.5871, 0.5661, 0.6270, 0.5930, 0.6801, 0.5242, 0.7265, and 0.3315, respectively.

However, there were still generally good cross-neutralization levels between the different genotypes/sub-genotypes and C4 in these sera. The observed differences in sera from naturally infected infants and children might be due to differences in previous infection history in some of these infants and children.

## 4. Discussion

The breadth of cross-neutralizing activities induced by EV71 vaccines is probably one of the the most important parameters for evaluating the effectiveness of vaccines against different genotypes and non-homologous viruses of the same genotype. In 2020, WHO issued recommendations for the quality, safety, and efficacy of enterovirus 71 vaccines (inactivated). A key highlight of the recommendations was the need to study and characterize the cross-neutralizing ability of antibodies induced by vaccines [17]. Similarly, the Key points of Quality Control and Evaluation of EV71 vaccine issued by China in 2016 also suggested that elicitation of a broad neutralizing activity should be a major consideration for the selection of a strain for the formulation of a vaccine. Several studies have investigated cross-antigenic properties of EV71 strains. A study by Mizuta et al. indicated that genotype B viruses could induce stronger cross-neutralizing antibody responses against B2, B4, B5, C1, C2, and C4 viruses than genotype C viruses did [37]. However, a different study found the opposite result [38]. Furthermore, results from studies conducted by Chia, et al. suggested that B and C EV71 viruses were equivalent in immunogenicity [39]. In view of the apparent differences shown in the literature, we characterized the neutralizing antibodies elicited by EV71 strains, including those from A, B0–B4, C1, C2, C4, and C5 genotypes/sub-genotypes. Sera from rats individually immunized with these 10 EV71 strains were employed to study cross-neutralization activities against these strains. The results indicated that A, B, and C genotype/sub-genotypes viruses could induce antibodies with an ability to neutralize strains from other genotypes/sub-genotypes. Based on NTAb GMTs against the different strains, the EV71 A strain was the most poorly neutralized by all sera except of that induced by the homologous strain. The NTAb GMT from all sera against it was 14.3, which was significantly lower than that against other EV71 sub-genotypes (*p* < 0.05). NTAb levels against B and C sub-genotypes also varied; NTAb values of rat sera inoculated with B4, C1, C2, and C4 viruses showed the most homogenous immune response against different EV71 sub-genotypes with MAX/MIN ratios between 4.0 and 6.0 (B4 = C1 < C4 < C2); NTAb MAX/MIN ratios induced by B0, B1, B2, and C5 viruses ranged between 10.7 and 16.0 while NTAb MAX/MIN ratios induced by A and B3 viruses were the highest, 256.0 and 48.0, respectively, suggesting higher specificity in their immune responses. Notably, the NTAb MAX/MIN ratio of sera from rats inoculated with the C4 vaccine strain was 5.3, indicating C4 sub-genotype possessing a good ability to induce cross neutralizing antibodies. The NTAbs of anti-B4 and anti-A against homologous immune strains were the lowest among all strains. The NTAbs may be obviously underestimated using A as a detection strain which was the most difficult to be neutralized. For B4, further studies on the correlation between biological characteristics with genes, epitopes, structures, as well as cross-antigen characteristics analysis of other B4 strains should be conducted to further understand and reveal the theoretical basis of the cross-antigen, cross-neutralization and other biological characteristics of EV71.

Other members of the Picornaviridae family associated with human disease show similar or higher sequence variability as EV71. Poliovirus is classified into three serotypes with different genetic clusters within each of the three serotypes. In addition, hepatitis A virus (HAV) consists of one serotype and four genotypes. Vaccines against poliovirus and HAV are made up of three serotypes and one serotype, respectively. Both vaccines have been on the market worldwide for decades since they were launched in 1955 and 1991, respectively, and have been shown to be effective against outbreaks caused by any poliovirus or hepatitis A virus [40,41,42]. Particularly, two of the three wild-type poliovirus serotypes haven eradicated globally and the remaining serotype 1 is only present in Pakistan and Afghanistan, which means global eradication is in sight with the concern that currently circulating vaccine-derived polioviruses would need to be eliminated as well. We are similarly hopeful that currently licensed C4 EV71 vaccines will be effective in preventing HFMD caused by different EV71 genotypes/sub-genotypes. The results of studies on a B4 sub-genotype EV71-based vaccine candidate showed a robust cross-neutralizing response in adults against EV71 sub-genotypes B1, B4, B5, and C4a and a marginally lower efficiency against C4b but failed to neutralize C2 EV71 [43]. This difference could be related to different antigenic characteristics between different EV71 strains [43,44]. The cross-neutralizing potential of NTAbs elicited by C4 EV71 vaccines have been studied against EV71 C2, C4, C5, B4, and B5 sub-genotypes [41]. However, the ability of the C4 EV71 based vaccines to confer protection against other EV71 genotypes/sub-genotypes was unknown. In this study, we tested sera of rats inoculated with the three approved EV71 vaccines as well as sera from infants and children immunized with the same vaccines for their cross-neutralizing ability. The results demonstrated that, excluding the A genotype, NTAb MAX/MIN ratios of sera from rats inoculated with the three vaccines were between 5.1 and 7.1, whereas NTAb MAX/MIN ratios of sera from immunized infants and children that were sero-negative or sero-positive before immunization were 1.8–3.4 and 1.8–2.4, respectively, showing similar values to those of sera from naturally infected individuals. These results suggested that the NTAb MAX/MIN ratios of sera from infants and children immunized with C4 EV71 vaccines were better than those observed in experimental animals in terms of producing a more consistent and homogenous and cross-reactive immune response. In addition, correlation between immune responses against the different EV71 genotypes/sub-genotypes and that against EV71 C4 in vaccinated infants and children was better than that observed in naturally infected infants and children. A NTAb against C4 EV71 between 1:16 and 1:32 was suggested as a correlate of vaccine protection in a phase III clinical study of a C4 EV71 vaccine [20]. Our results could provide a reference for estimating neutralizing antibody protective thresholds against other EV71 genotypes/sub-genotypes. Results from our study show that BrCr, the EV71 genotype A prototype strain, which has not been associated with outbreaks anywhere in the world for many years, is the most poorly neutralized strain by both sera from immunized rats and infants and children. Phylogenetic trees generated with P1 capsid genomic sequences showed the genetic distance between both B and C genotypes and BrCr strain (Figure S2). We analyzed differences in the genotypes/sub-genotypes at key amino acid sequences among the different EV71 strains used in the study in order to correlate sequence differences to differences in cross-neutralization activities observed for the different EV71 genotypes/sub-genotypes. Interestingly, when neutralizing epitopes were aligned, sequences at amino acids VP1-167, VP1-172, and VP2-143 were found to be distinct for the genotype A virus (Table S1, Figure S3). An aspartic acid was found at position 167 of VP1 in genotype A, replaced by glutamic acid in the other viruses. Although both residues are acidic and their electrostatic potential is positive, the side chain of glutamic acid is longer and could create steric obstructions for the binding of antibodies. The position 172 of VP1 in genotype A is occupied by proline, a non-polar amino acid. In contrast, a polar amino acid, glutamine, is observed at this position in other EV71 sub-genotypes. The hydrophobicity of the former is stronger than the latter, and an α-helix exists here, which could be influenced by the nature of amino acids in the vicinity. Another significant difference is observed at position 143 of VP2. The amino acid in A at this position is asparagine, which is polar in nature. It is replaced by an aspartic acid in other sub-genotypes. Both these amino acids differ in electrostatic potential and this difference could impact the interaction of the antigen with the antibodies. The overall changes in the amino acids could impact the local 3-D configuration, which probably has an effect on the cross-neutralizing activity. Determination of the structures of the various sub-genotypes of EV71 and comparison of the structure of their epitopes could shed further light on the differences in susceptibilities of these viruses to cross-neutralization.

In conclusion, we found that the 10 sub-genotypes of EV71 tested in this study could induce cross-neutralizing antibodies with varying efficiencies; the cross-neutralization of genotype A was the least efficient. The three commercially developed C4 EV71 vaccines elicited good cross-neutralizing activities against all genotypes/sub-genotypes of EV71 in both experimental animals and infants and children. However, as EV71 continues to evolve, a combination of immuno-surveillance and testing of the NTAbs elicited by the currently available vaccines for their ability to cross-neutralize these new strains needs to be constantly monitored. Identification of conserved epitopes across the sub-genotypes, testing their immunogenecity and incorporating them in vaccines could ensure pan-EV71 protection.

## Figures and Tables

**Table 1:** List of EV71 sub-genotypes used in the study.

Sub-Genotype	Name	Year	Country	Genbank No.	Mean of Virus Titer (Lg CCID/mL)
A	BrCr/A	1970	USA	U22521.1	7.69
B0	66-10857/B0	1966	Netherlands	AB524084	9.53
B1	71-17000/B1	1971	Netherlands	AB524110	9.13
B2	86-11316/B2	1986	Netherlands	AB524132	8.84
B3	MAL-97-B3/B3	1997	Malaysia	JN874550	8.53
B4	JPN-97-B4/B4	1997	Japan	LC375765	8.84
C1	91-480/C1	1991	Netherlands	AB524200	9.5
C2	36-92/C2	2007	Netherlands	AB524268	9.25
C4	523-07T/C4		China	EU753398.2	8.88
C5	E200525-TW/C5	2006	Japan	/	8.72

**Table 2:** Neutralization activities of serum samples obtained from rats challenged with 10 sub-genotypes of EV71 individually.

Sub-Genotype	Serum Sample										GMTs
	Anti-A	Anti-B0	Anti-B1	Anti-B2	Anti-B3	Anti-B4	Anti-C1	Anti-C2	Anti-C4	Anti-C5	
A	**64**	4	96	16	4	48	4	4	12	24	14.3 *
B0	64	**128**	384	512	8	24	24	24	48	1024	78.4 **
B1	384	32	**512**	384	16	32	16	16	48	96	65.5 ***
B2	1536	24	128	**1536**	96	32	16	48	48	512	115.4
B3	8192	96	512	1024	**192**	24	16	32	256	128	178
B4	16,384	256	768	6144	384	**8**	48	64	256	192	313.5 ^#^
C1	256	48	1536	768	128	24	**24**	64	64	1024	146.3
C2	128	64	1536	1024	48	32	24	**96**	96	1024	146.3
C4	1024	48	192	768	48	32	12	24	**192**	256	104.7
C5	192	32	1024	512	64	32	16	32	64	**1024**	108.3 ^##^
MAX/MIN (with A)	256	64	16	384	96	6	12	24	21.3	42.7	22
MAX/MIN (without A)	256	10.7	12	16	48	4	4	6	5.3	10.7	4.8

*: The differences between A genotype and other sub-types were significant. **: The differences between B0 sub-genotype and C2 were significant. ***: The differences between B1 sub-genotype and B3, B4, C1, C2 sub-types were significant. ^#^: The differences between B4 sub-genotype and B2, B3, C4 sub-types were significant. ^##^: The differences between C5 genotype and C1 sub-types were significant. The bold values were NTAbs of anti-sera and against homologous immune strains.

**Table 3:** Neutralizing antibody titers of serum samples from vaccinated rats against 10 Sub-genotypes of EV71.

Sub-Genotypes	Vaccines			GMTs
	A	B	C	
A	37.4 (13.8–101.0)	172.9 (36.8–811.8)	805.7 (486.3–1334.9)	173.4 *
B0	176.0 (34.4–899.2)	1627.0 (199.2–13,288.9)	2390.9 (829.4–6892.1)	881.4
B1	156.8 (41.0–598.8)	1204.3 (144.4–10,043.6)	2390.9 (943.0–6061.8)	767.2
B2	237.2 (38.9–1446.3)	1137.0 (97.4–13,269.4)	3478.3 (962.3–12,572.4)	978.9
B3	284.9 (76.0–1068.3)	1137.0 (132.0–9794.6)	717.8 (324.0–1590.3)	614.9
B4	395.0 (93.3–1672.3)	1137.0 (132.0–9794.6)	1315.7 (513.2–3373.4)	839.1
C1	627.1 (138.2–2846.2)	2273.9 (332.4–15,553.6)	2088.6 (906.0–4815.0)	1438.8 **
C2	313.5 (50.4–1950.9)	1416.4 (132.7–15,121.1)	1690.6 (681.9–4191.3)	908.8
C4	88.0 (22.5–344.4)	841.6 (60.5–11,708.5)	1219.1 (528.9–2809.9)	448.6 ***
C5	522.2 (98.9–2756.8)	5325.2 (380.0–74,634.8)	3649.1 (1243.7–10,707.0)	2165
Max/Min (with A)	16.8	30.8	5.1	-
Max/Min (without A)	7.1	6.3	5.1	-

*: The differences between A genotype and B0, B1, B2, C5 were significant when put all three vaccine immune sera together. **: The differences between C1 sub-genotype and B3, B4 were significant when put all three vaccine immune sera together. ***: The differences among C4 sub-genotype and B0, B1, C5 were significant when put all three vaccine immune sera together.

**Table 4:** Cross-neutralizing antibodies induced by three C4 EV-A71 vaccines in infants and children.

Sub-Genotype	Neutralizing Antibody Titer Induced by						Naturally Infected Serum
	Vaccine A		Vaccine B		Vaccine C		
	Prevaccination Sero-Negative	Prevaccination Sero-Positive	Prevaccination Sero-Negative	Prevaccination Sero-Positive	Prevaccination Sero-Negative	Prevaccination Sero-Positive	
A	15.3 (11.1–21.1)	113.1 (88.0–145.4)	32.0 (24.0–42.8)	183.8 (122.8–275.1)	42.8 (33.8–54.1)	205.1 (152.2–276.5)	40.8 (31.9–52.2)
B0	118.5 (82.5–170.2)	1306.0 (1005.0–1697.2)	199.8 (146.5–272.5)	2268.0 (1257.1–4092.0)	328.4 (237.4–454.4)	2681.3 (1890.9–3802.0)	318.1 (210.3–481.2)
B1	120.5 (78.8–184.4)	1379.8 (1081.7–2219.7)	235.6 (158.9–349.4)	2544.6 (1561.5–4146.7)	432.0 (322.3–579.0)	3151.3 (2416.3–4109.9)	382.7 (217.1–674.8)
B2	170.0 (100.1–288.8)	956.1 (748.2–1221.8)	292.5 (202.3–423.0)	3044.0 (1611.7–5749.0)	549.1 (352.3–855.9)	3715.8 (2515.2–5489.6)	427.2 (258.2–706.9)
B3	195.3 (135.3–281.8)	701.7 (523.2–941.2)	242.5 (164.4–357.7)	1862.8 (1219.1–2846.3)	352.0 (236.0–525.0)	2923.0 (2232.3–3827.4)	256.2 (171.6–382.5)
B4	237.6 (164.9–342.4)	772.0 (586.4–1016.4)	355.0 (251.5–501.1)	2388.2 (1480.2–3853.4)	475.2 (323.4–698.4)	3747.5 (2646.3–5307.1)	369.7 (235.6–580.2)
C1	227.6 (167.7–308.8)	1091.8 (816.4–1460.1)	331.2 (214.4–511.8)	3589.3 (2233.1–5769.0)	485.0 (322.5–729.3)	4874.3 (3646.6–6515.3)	487.8 (337.7–704.6)
C2	179.6 (121.2–266.2)	1287.4 (965.2–1717.1)	292.5 (196.4–435.7)	2923.0 (1716.7–4977.0)	422.2 (314.9–566.0)	2799.5 (2166.1–3618.2)	370.7 (249.1–551.6)
C4	296.8 (213.3–413.0)	1513.0 (1110.0–2062.4)	284.2 (201.1–401.7)	2354.1 (1377.5–4023.2)	304.6 (212.1–437.6)	2743.4 (2116.3–3556.2)	313.5 (202.5–485.5)
C5	400.7 (265.3–605.2)	1688.7 (1251.2–2279.3)	290.1 (171.7–490.1)	3000.5 (1559.4–5773.2)	391.6 (246.8–621.3)	4613.8 (3773.4–5641.3)	724.6 (428.0–1226.7)
MAX/ MIN (with A)	26.2	14.9	11.1	19.5	12.8	23.8	17.8
MAX/MIN (without A)	3.4	2.4	1.8	1.9	1.8	1.8	2.8

Note: Data are GMT (95% CI). GMT means geometric mean titer.

## Data Availability

The data presented in this study are available on request from the corresponding author.

## Supplementary Materials

The following are available online at https://www.mdpi.com/article/10.3390/v13050720/s1, Figure S1: The MAX/MIN of 10 sera against viruses with and without A. Figure S2: Phylogenetic tree of EV71 strains based on partial P1 coding sequences. Figure S3: The P1 sequences of ten viruses. Table S1: Neutralizing sites analysis of 10 EV71 sub-genotype strains of P1 region.
