# Supplementary material for: Cross-Antigenicity between EV71 Sub-Genotypes: Implications for Vaccine Efficacy
Source: Viruses. 2021 Apr 21;13(5):720. doi: 10.3390/v13050720 (PMC8143144; doi:10.3390/v13050720)
Supplement: Supplementary file 1 [file viruses-13-00720-s001.zip › viruses-1177268-supplementary.pdf]

viruses

Supplementary Materials: Cross-Antigenicity between EV71 Sub-Genotypes: Implications for Vaccine Efficacy

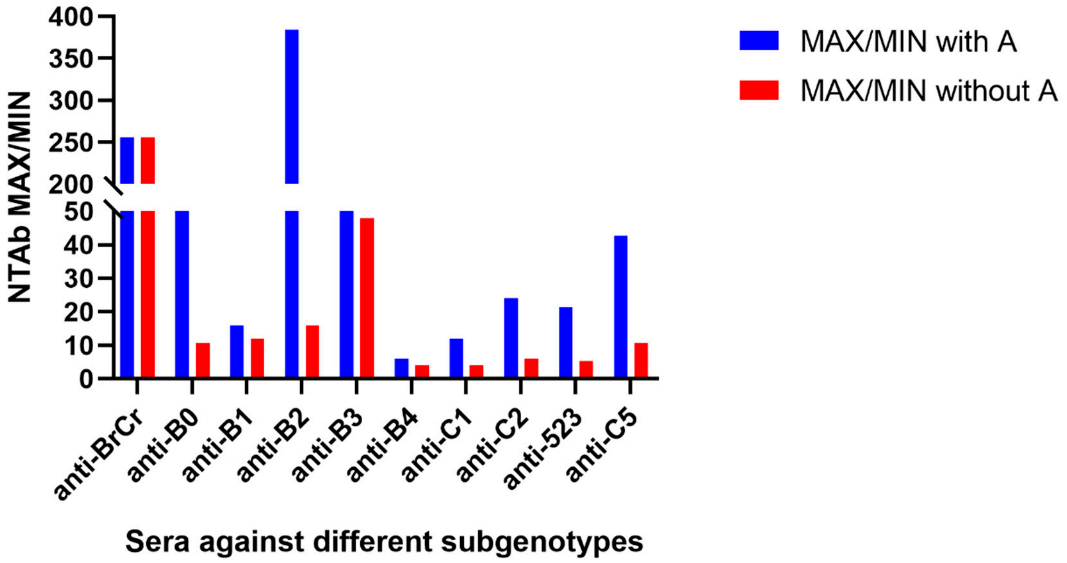

Figure S1. The MAX/MIN of 10 sera against viruses with and without A

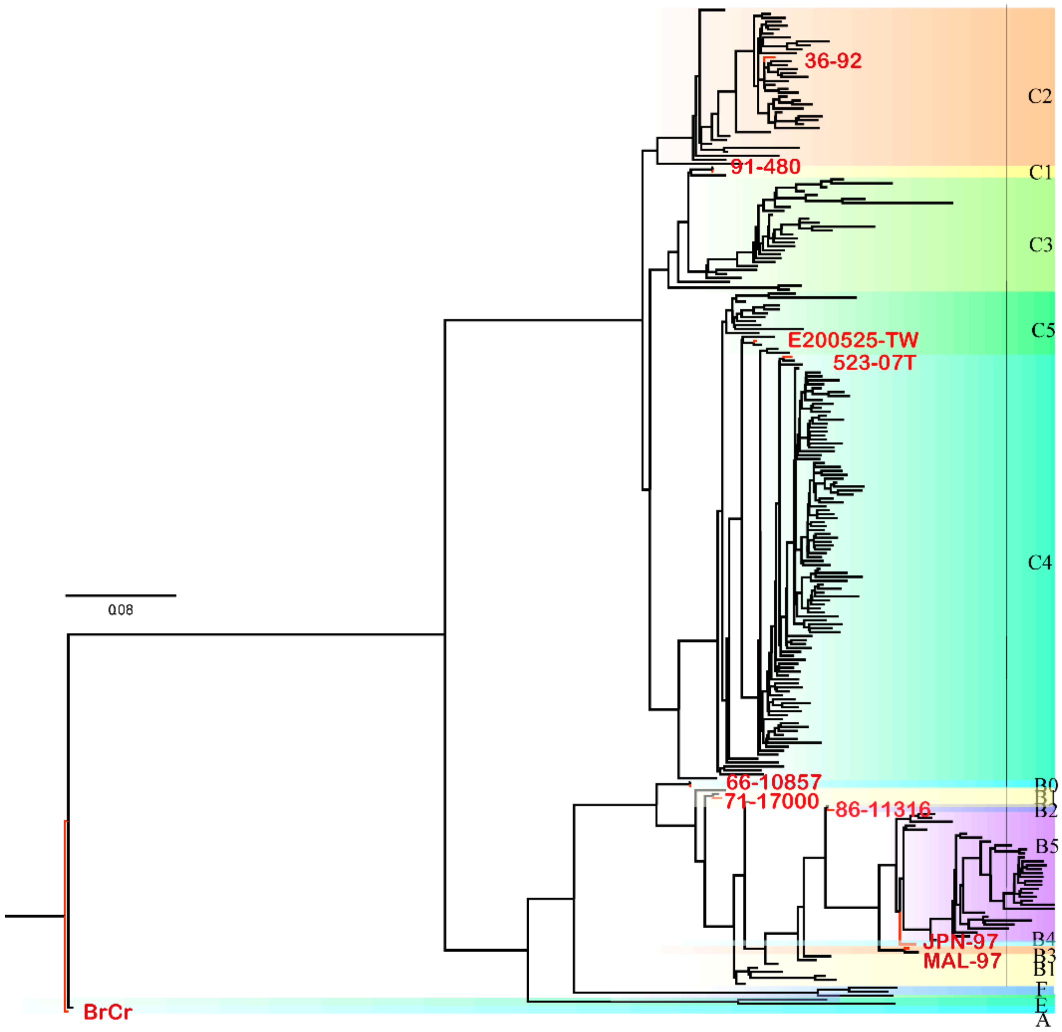

Figure S2. Phylogenetic tree of EV71 strains based on partial P1 coding sequences

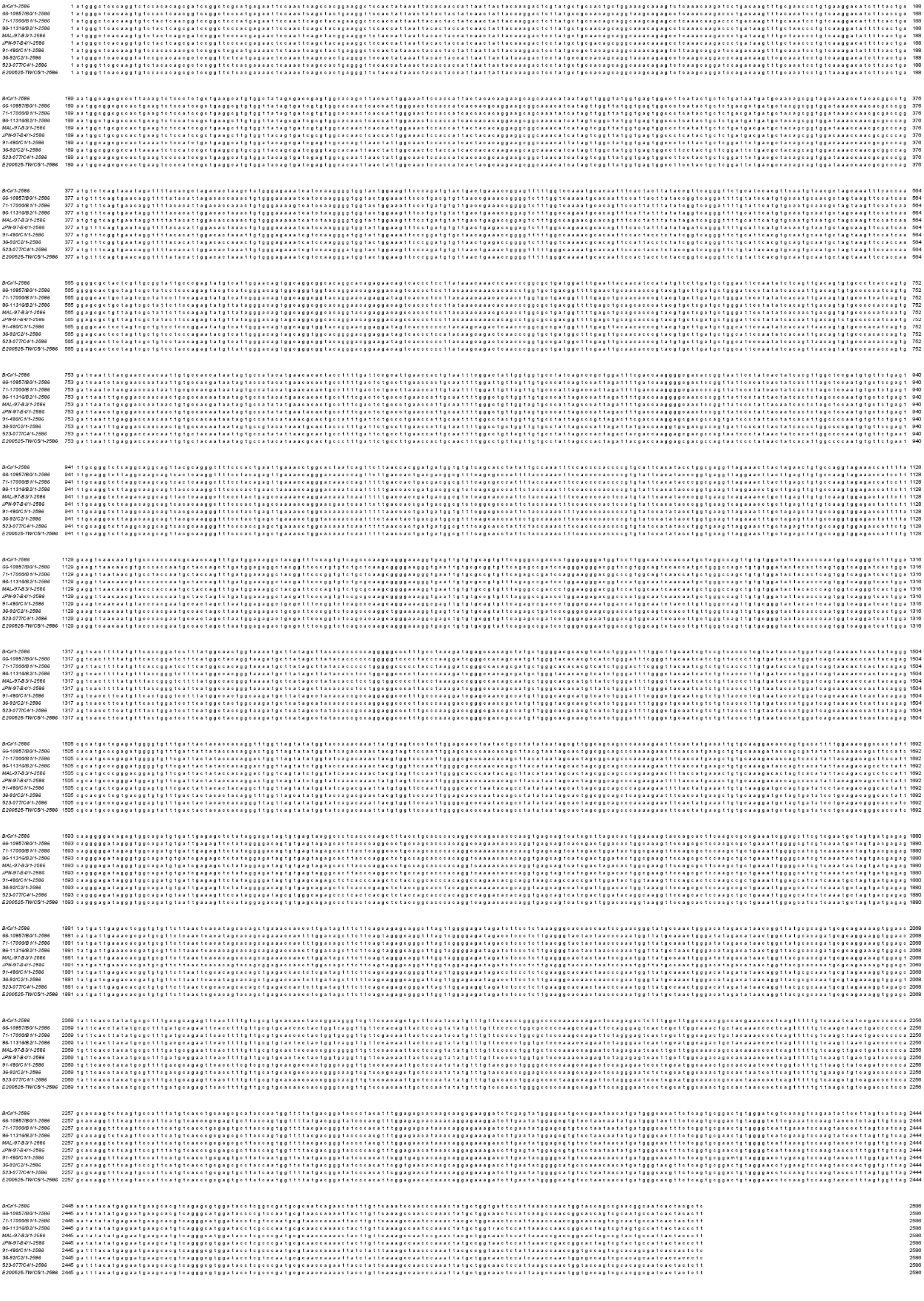

Figure S3. The P1 sequences of ten viruses.

Table S1. Amino acid sequences at key positions in EV71 strains from different sub-genotypes.

**Strain**	**VP1**							**VP2**	**VP4**
	43	98	145	148	164	167	172	143	7
A	K	K	R	P	D	***D***	***P***	***N***	T
B0	K	E	Q	P	D	E	Q	D	T
B1	E	N	E	R	D	E	Q	D	T
B2	E	E	Q	P	E	E	Q	D	T
B3	E	E	G	P	E	E	Q	D	T
B4	E	E	E	P	E	E	Q	D	T
C1	K	E	Q	P	D	E	Q	D	T
C2	K	E	Q	P	D	E	Q	D	A
C4	K	E	Q	P	D	E	Q	D	T
C5	K	E	Q	P	D	E	Q	D	T
